# Synthetic mRNA‐based differentiation method enables early detection of Parkinson's phenotypes in neurons derived from Gaucher disease‐induced pluripotent stem cells

**DOI:** 10.1002/sctm.20-0302

**Published:** 2020-12-20

**Authors:** Tomohiko Akiyama, Saeko Sato, Shigeru B. H. Ko, Osamu Sano, Sho Sato, Masayo Saito, Hiroaki Nagai, Minoru S. H. Ko, Hidehisa Iwata

**Affiliations:** ^1^ Department of Systems Medicine Keio University School of Medicine Tokyo Japan; ^2^ Neuroscience Drug Discovery Unit, Research Takeda Pharmaceutical Company Limited Fujisawa Japan; ^3^ DMPK Laboratories, Research Takeda Pharmaceutical Company Limited Fujisawa Japan

**Keywords:** differentiation, induced pluripotent stem cells (iPSCs), neural differentiation, Parkinson's disease, transcription factors

## Abstract

Gaucher disease, the most prevalent metabolic storage disorder, is caused by mutations in the glucocerebrosidase gene *GBA1*, which lead to the accumulation of glucosylceramide (GlcCer) in affected cells. Gaucher disease type 1 (GD1), although defined as a nonneuronopathic subtype, is accompanied by an increased risk of Parkinson's disease. To gain insights into the association of progressive accumulation of GlcCer and the Parkinson's disease phenotypes, we generated dopaminergic (DA) neurons from induced pluripotent stem cells (iPSCs) derived from a GD1 patient and a healthy donor control, and measured GlcCer accumulation by liquid chromatography‐mass spectrometry. We tested two DA neuron differentiation methods: a well‐established method that mimics a step‐wise developmental process from iPSCs to neural progenitor cells, and to DA neurons; and a synthetic mRNA‐based method that overexpresses a transcription factor in iPSCs. GD1‐specific accumulation of GlcCer was detected after 60 days of differentiation by the former method, whereas it was detected after only 10 days by the latter method. With this synthetic mRNA‐based rapid differentiation method, we found that the metabolic defect in GD1 patient cells can be rescued by the overexpression of wild‐type GBA1 or the treatment with an inhibitor for GlcCer synthesis. Furthermore, we detected the increased phosphorylation of α‐synuclein, a biomarker for Parkinson's disease, in DA neurons derived from a GD1 patient, which was significantly decreased by the overexpression of wild‐type GBA1. These results suggest that synthetic mRNA‐based method accelerates the analyses of the pathological mechanisms of Parkinson's disease in GD1 patients and possibly facilitates drug discovery processes.

## Graphical abstract

Synthetic mRNA‐based expression of transcription factors enhanced induced pluripotent stem cell differentiation and enabled early (10 days) detection of a risk factor of Parkinson's disease, i.e. the accumulation of glucosylceramide (GlcCer) in Gaucher patient neurons. This rapid differentiation method also enabled to detect the phosphorylation of α‐synuclein—a critical Parkinson's disease‐linked phenotype.

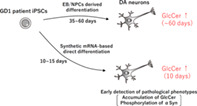

**Significance statement**

Gaucher disease type 1 (GD1) is associated with an increased risk of developing Parkinson's disease. Induced pluripotent stem cells (iPSCs) derived from the patients can be useful for pathological analyses. However, the commonly used method for neuron differentiation is complex and requires 35 to 60 days before the formation of mature neurons. This study demonstrated that synthetic mRNA‐based expression of transcription factors enhanced iPSC differentiation and enabled early detection (10 days) of a risk factor of Parkinson's disease, that is, the accumulation of glucosylceramide (GlcCer), in mature neurons. These results suggest that synthetic mRNA‐based method accelerates the analyses of the pathological mechanisms of Parkinson's disease in GD1 patients and possibly facilitates drug discovery processes.

## 1 INTRODUCTION

Gaucher disease is an inherited metabolic storage disorder, which is caused by the mutations of *GBA1*, a gene encoding lysosomal enzyme β‐glucocerebrosidase. GBA1 enzyme is responsible for the catalytic degradation of glucosylceramide (GlcCer) into glucose and ceramide to maintain cellular homeostasis, mainly in the monocyte/macrophage system.^1^, ^2^, ^3^ Accumulation of GlcCer due to the deficiency of this enzyme interferes with an autophagy‐lysosomal pathway, resulting in impaired turnover of organelles and proteins, including the accumulation of dysfunctional mitochondria.^4^, ^5^, ^6^ The most prevalent type of Gaucher disease is type 1 (GD1), which is distinguished from type 2 and type 3 by the absence of complications in the central nervous system. Patients with GD1 carry the specific mutations of *GBA1*,^7^, ^8^ which may preclude the development of primary neuronopathic disease. The major clinical symptoms of GD1 patients are pancytopenia, hepatosplenomegaly, and bone disease.^9^, ^10^

However, cohort studies demonstrated that GD1 is highly associated with an increased life‐time risk of developing Parkinson's disease.^11^ Parkinson's disease is characterized pathologically by dopaminergic neurodegeneration with Lewy body inclusions, composed of phosphorylated α‐synuclein aggregates.^12^, ^13^, ^14^ Recent reports revealed that mutated GBA1 enzyme is associated with lysosomal α‐synuclein and compromises its degradation, which causes a gradual accumulation of α‐synuclein oligomers.^15^, ^16^, ^17^ Moreover, biochemical analysis showed that GlcCer affects in vitro formation of α‐synuclein fibrils and soluble oligomers.^15^, ^18^ Therefore, the gradual accumulation of GlcCer is considered as a risk factor of developing the Parkinson disease.

To better understand the pathogenesis of neuronal defects in GD1 patients, induced pluripotent stem cells (iPSCs) derived from patients may be a useful resource for their unlimited cell supply and ability to differentiate into dopaminergic (DA) neurons in vitro.^19^ However, the commonly used method for DA neuron differentiation is complex and requires 35 to 60 days before the formation of functional neurons. The method includes several intermediate steps, such as embryoid body formation, induction of neuronal precursor cells (NPCs), and neuronal specification. Those problems hamper the production of large quantities of homogenous neurons for pathological analysis and drug screening.

As an alternative approach, the overexpression of neurogenic transcription factors (TFs), such as PITX3, ATOH1, ASCL1, NURR1, or LMX1A, have been used to directly differentiate DA neurons from iPSCs.^20^, ^21^, ^22^ However, it is not well studied whether the directly differentiated DA neurons display the same pathological phenotypes as those induced by the conventional method. Also, commonly used methods of delivering TFs to cells via plasmids, lentiviruses, or adeno‐associated virus vectors usually require cumbersome steps of isolating iPSC lines with the TF‐integrated into the genome.

We have previously developed an efficient method to differentiate human iPSCs by introducing synthetic mRNA (synRNA) encoding TFs.^23^, ^24^, ^25^, ^26^, ^27^ Compared with the DNA‐based approaches such as plasmids and viruses, the transfection of synthetic mRNAs is efficient enough to facilitate the direct differentiation of human iPSCs. In this study, we applied the mRNA‐based method to generate DA neurons from GD1 patient iPSCs, and compared the timing of GlcCer accumulation with that of the conventional differentiation method. We also tested whether the pathological phenotypes can be rescued by the overexpression of wild‐type GBA1 or by the treatment of iPSCs with an inhibitor of GlcCer synthesis.

## 2 MATERIALS AND METHODS

### 2.1 Generation of iPSC lines and culture

To generate GD1 patient‐derived iPSCs, skin fibroblasts of the patient (29 years, male, GM00372) were obtained from the NIGMS Human Genetic Cell Repository at the Coriell Institute for Medical Research. The donor subject is a compound heterozygote for the following mutations: one allele carries a single‐base mutation (1226A>G) in the *GBA* gene, which results in the amino acid substitution of serine for asparagine (N370S); the second allele carries an insertion of a second guanine at cDNA nucleotide 84 (84GG). Three iPSC clones (clones 1, 2, 3) from the same donor were generated with StemRNA‐NM reprogramming kit (Stemgent) at ReproCELL, Japan. As the healthy control, 201B7 iPSCs were obtained from CiRA (Kyoto, Japan).^28^ To rescue the enzymatic defect of GBA1, wild‐type GBA1 cDNA was cloned into the PiggyBac expression vector and the tet‐inducible iPSC line was generated as described previously.^29^ Undifferentiated iPSCs were maintained in StemFit AK‐02 medium (Ajinomoto, Tokyo, Japan) on iMatrix‐511 (Nippi, Tokyo, Japan)‐coated plates.

### 2.2 Differentiation of iPSCs into DA neurons (conventional method)

DA neurons were generated from iPSCs based on a previously published method^30^ with some modifications. To induce dopaminergic NPCs, iPSCs were cultured on Matrigel‐coated plates in Neurobasal/B‐27 medium (NB medium) supplemented with 2 mM l‐glutamine, 0.5 μM LDN193189 (AXON MedChem, Reston, Virginia), 1 μM CHIR99021 (Wako, Osaka, Japan), 10 μM SB431542 (Wako), 200 ng/mL Sonic hedgehog (SHH) (R&D Systems, Minneapolis, Minnesota), and 0.5 μM Purmorphamine (Wako) for 5 days. Then the cells were cultured in NB medium supplement with 0.5 μM LDN193189 and 1 μM CHIR99021 for 7 days. The medium was changed every other day. Twelve days after the start of differentiation, NPCs are passaged on laminin (R&D)‐coated plates and cultured for 7 days in Floor Plate Cell Expansion medium (FPE medium, Gibco Thermo Fisher Scientific, Waltham, Massachusetts) to promote proliferation. To generate neurosheres, NPCs were detached and grown on low‐binding plates (Corning, New York) with FPE medium for 5 days. The spheres were then treated with Accutase (Innovative Cell Technologies, San Diego, California) and seeded on Matrigel‐coated plates for differentiation into DA neurons. Finally, the cells were cultured in NB medium supplemented with 2 mM l‐glutamine, 100 μM ascorbic acid (Sigma‐Aldrich, St Louis, Missouri), 0.5 mM dbcAMP (Sigma), PD0325901 (Wako), and Activin A (R&D) for ~14 days.

### 2.3 Differentiation of iPSCs into DA neurons (RNA‐based induction method)

The modified mRNA encoding NGN2 was synthesized as previously described.^25^ RNA transfection was performed with Lipofectamine Messenger Max (Invitrogen Thermo Fisher Scientific), according to the manufacturer's instructions. Briefly, 1 μg mRNA in OptiMEM (Gibco) was mixed with 2 μL transfection reagent in OptiMEM and incubated for 5 minutes for complex formation. Complexes were then added dropwise to wells. For differentiation, iPSCs were seeded on iMatrix‐511 (Nippi)‐coated plates and cultured in StemFit AK‐02 medium containing Y27632. The next day, synthetic mRNAs were transfected into iPSCs twice with an interval of 5 hours, followed by another transfection on day 2. The medium was replaced 3 hours post each transfection. To induce the midbrain dopaminergic lineage, the cells were cultured in N2B27 medium supplemented with 100 nM LDN193189, 10 μM SB431542, 100 ng/mL SHH, and 100 ng/mL fibroblast growth factor 8 (FGF8) for 4 days. The cells were then cultured in N2B27 medium supplemented with 20 ng/mL BDNF (R&D), 0.2 mM ascorbic acid (Sigma), 20 ng/mL GDNF (R&D), 1 ng/mL TGFβ3, 0.5 mM dbcAMP (Sigma), and 10 μM DAPT for 9 days to promote differentiation. To remove undifferentiated cells, 3 μg/mL aphidicolin was added from day 3 to day 5 postdifferentiation. In some experiments, the differentiated cells were treated with d,l‐threo‐1‐Phenyl‐2‐palmitoylamino‐3‐morpholino‐1‐propanol (PPMP) (Sigma) or doxycycline (1 μg/mL) from day 3 postdifferentiation.

### 2.4 Quantification of GlcCer concentration

The cells were extracted with a 1:1 mixed solution of 2‐propanol and ethanol. After centrifugation, the supernatants were used for liquid chromatography‐mass spectrometry (LC/MS) analysis. The LC/MS was performed with an API‐4000 or API‐5000 triple‐quadrupole mass spectrometer (AB Sciex, Tokyo, Japan) interfaced with Prominence UFLC system (Shimadzu, Kyoto, Japan). The concentration of GlcCer and Cer was measured as previously described.^31^, ^32^ The two GlcCer isoforms (C16:0 and C24:1) were analyzed.

### 2.5 Real‐time quantitative reverse transcription PCR

Total RNA was isolated with TRIzol reagent (Invitrogen) or RNeasy Mini Kit (Qiagen, Hilden, Germany), and cDNAs were generated with random hexamers using the PrimeScript RT Master Mix (Takara, Shiga, Japan) or the ReverTra Ace kit (Toyobo, Osaka, Japan). Real‐time PCR was performed using SYBR Green PCR system (Takara) or TaqMan PCR system (Applied Biosystems, Foster City, California). The primer sequences used were as follows: MAP2, 5′‐ccaatggattcccatacagg‐3′ and 5′‐tctccgttgatcccattctc‐3′, tyrosine hydroxylase (TH), 5′‐ccgtgctaaacctgctcttc‐3′ and 5′‐atggtggattttggcttcaa‐3′, DAT, 5′‐ttcatcatctacccggaagc‐3′ and 5′‐ggtgagcagcatgatgaaga‐3′, EN1, 5′‐aagccacaggcatcaagaac‐3′ and 5′‐ctcgctctcgtctttgtcct‐3′, GIRK2, 5′‐tagaggacccctcctggact‐3′ and 5′‐atctgtgatgacccggtagc‐3′, AADC, 5′‐gagctgggttaattggtgga‐3′ and 5′‐gtctctctccagggcttcct‐3′.

### 2.6 Microarray analysis

Microarray data showing expression profiles during DA neuron differentiation^30^ were obtained from the Gene Expression Omnibus (Accession no. GSE32658). The gene expression profile matrix was generated with the ExAtlas, an online software tool for meta‐analysis and visualization of gene expression data.^33^ Average log‐expression value (log_10_) was calculated and compared at each time point from day 0 to day 25 postdifferentiation.

### 2.7 Immunostaining

The cells were fixed in 4% paraformaldehyde and permeabilized in 0.4% to 0.5% Triton X‐100. The cells were blocked with goat/bovine serum in phosphate‐buffered saline (PBS) and incubated for 3 hours or overnight with the following antibodies, OCT4 (Abcam, Cambridge, Massachusetts; ab19857), NANOG (Abcam ab21624), TH (Abcam; ab76442), TUJ1 (Cell Signaling, Danvers, Massachusetts; 5568S), DAT (GeneTex, Irvine, California; 133152), α‐synuclein (BD Biosciences Clontech, Palo Alto, California; 610787). After several washes in PBS, the samples were incubated with Alexa Fluor‐conjugated secondary antibodies; 488‐labeled goat anti‐rabbit IgG (Invitrogen, A11008) for the detection of OCT4, 488‐labeled goat anti‐chicken IgY (Abcam; ab150169) for TH, 568‐labeled goat anti‐rabbit IgG (Molecular Probes, Carlsbad, California, A11036) for TUJ1, and 594‐labeled goat anti‐rabbit IgG (Invitrogen, A11012) for NANOG and DAT, 488‐labeled goat anti‐mouse IgG (Invitrogen, A11001) for α‐synuclein. Nuclei were counterstained with DAPI (Dako, Hamburg, Germany) or NucSpot Live 650 Nuclear Stain (Biotium, Hayward, California). Immunofluorescence was visualized with an inverted fluorescence microscope IX73 (Olympus, Tokyo, Japan) or a confocal microscope Cell Voyager 7000 (Yokogawa, Tokyo, Japan). The fluorescence intensities were quantified using CV7000 analysis software.

### 2.8 Western blotting

The cells were lysed with a sample buffer (50 mM Tris‐HCl, pH 6.8, 2% SDS, 6% 2‐mercaptoethanol and 500 mg/mL urea). The proteins were separated by SDS‐PAGE on a 4‐15% polyacrylamide gel (Bio‐Rad, Hercules, California) and were electrically transferred to polyvinylidene difluoride membranes (Bio‐Rad). The membranes were blocked for 1 hour in Tris‐buffered saline containing 0.1% Tween‐20 (TBST) and 5% skimmed milk. The membranes were washed in TBST and then incubated with primary antibodies in TBS/2% BSA (1:1000) overnight at 4°C. The following antibodies were used; α‐Syn (GeneTex, GTX112799), S129‐P‐α‐Syn (Fujifilm, Tokyo, Japan, 015‐25191), Y125‐P‐α‐Syn (Abcam, ab10789), and β‐actin (Cell Signaling, 4970S). The membranes were washed and then incubated with horseradish peroxidase‐conjugated secondary antibodies (GE Healthcare, Chicago, Illinois) (1:2000) for 1 hour at RT. The membranes were washed in TBST, and immunoreactivity was visualized using an ECL Prime Detection Kit (GE Healthcare) and detected using a Luminescent Image Analyzer (LAS‐4000; Fujifilm). Immunoblots were quantified using ImageJ software. The signal intensities were normalized to the loading controls, and the average values were calculated from three independent experiments.

### 2.9 Statistical analysis

All results of experiments performed independently and in triplicate are presented as the mean and SD. Student's *t* test was used to analyze two unpaired groups. Statistical significance was established at *P* < .05.

## 3 RESULTS

### 3.1 Accumulation of GlcCer at the late stage of dopaminergic neuron differentiation

**FIGURE 1:**
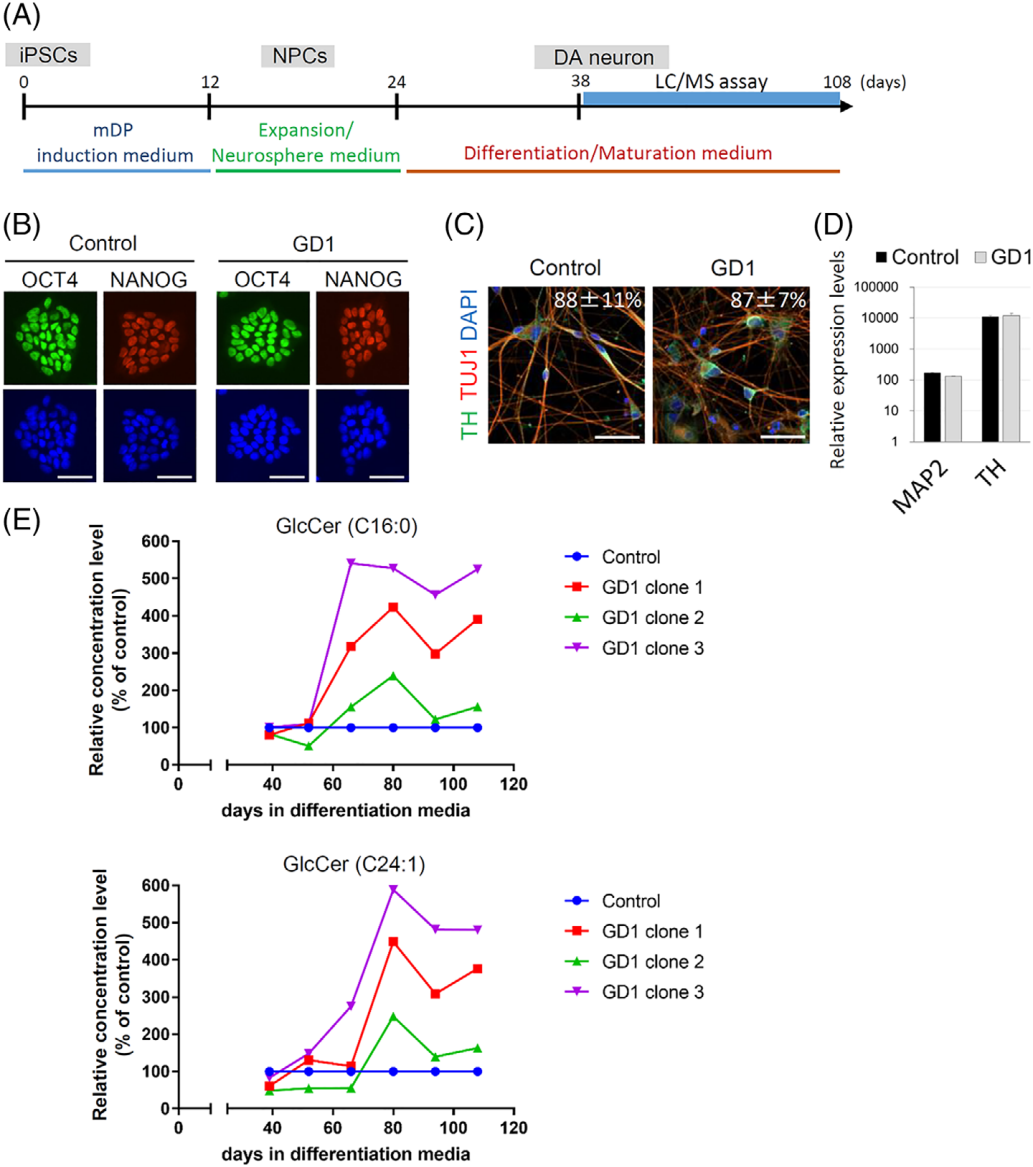
Temporal changes in glucosylceramide (GlcCer) concentration during dopaminergic differentiation of induced pluripotent stem cells (iPSCs) induced by the conventional method. A, Experimental scheme of iPSC differentiation into dopaminergic (DA) neurons. iPSCs were cultured in midbrain DA progenitor (mDP) induction medium. Neuronal progenitor cells (NPCs) were obtained after 12 days of differentiation. NPCs were expanded and neurospheres were formed at day 24 of differentiation. DA neurons were generated at day 38 of differentiation and further cultured for maturation. The medium components are described in Section 2. Liquid chromatography‐mass spectrometry (LC/MS) assay for GlcCer concentration was conducted from day 38 to day 108. B, Immunostaining analysis for pluripotency markers, OCT4 and NANOG in Gaucher disease type 1 (GD1) patient iPSCs and control iPSCs (201B7). Scale bar, 50 μm. C, Immunostaining analysis for neuronal markers in GD1 patient and control neurons. TUJ1 is a marker for general neurons and TH for DA neurons. Scale bar, 50 μm. Percentages of TH+ cells/TUJ1+ cells are shown. Mean ± SD (N = 3). D, Quantitative real‐time PCR analysis for neuronal markers in GD1 patient and control neurons. MAP2 labels for mature neurons and TH for DA neurons. The expression levels are normalized to iPSCs expression levels (iPSCs = 1). E, LC/MS analysis for temporal changes in GlcCer concentration during maturation of DA neurons. Two GlcCer isoforms (C16:0 and C24:1) were analyzed. The concentration levels relative to the control were shown. GD1, GD1 patient neurons; control, 201B7 neurons. The three iPSC clones (clones 1, 2, 3) from the same donor were analyzed

It is known that GlcCer is accumulated by defects in enzyme β‐glucocerebrosidase encoded by *GBA1*. However, the timing of accumulation during maturation of DA neurons has not been clarified. To investigate temporal changes in the GlcCer concentration, we first differentiated GD1 patient‐derived iPSCs into DA neurons based on the previous protocol^30^ with some modifications (Figure 1A, see also details in Section 2). A healthy donor‐derived iPSCs (201B7)^28^ were used as a control and differentiated in the same condition. On the undifferentiated condition, both patient and control cells expressed pluripotent cell markers (Figure 1B). After 38 days of differentiation, most cells were positive for a marker of DA neurons—TH (Figure 1C). The differentiation efficiencies were not different between patient and control cells (Figure 1C,D). We quantified the temporal changes in GlcCer, starting from day 38 by LC/MS. From day 38 to day 50, GlcCer concentration in patient cells was not different from control cells (Figure 1E). However, when DA neurons were further cultured for maturation (day 60 to day 80), the concentration of GlcCer was significantly increased in GD1 patient cells compared with control cells (Figure 1E). These results suggest that GlcCer is specifically accumulated at the late maturation stages in the patient‐derived iPSCs.

### 3.2 Direct differentiation of DA neurons from GD1 patient‐derived iPSCs

**FIGURE 2:**
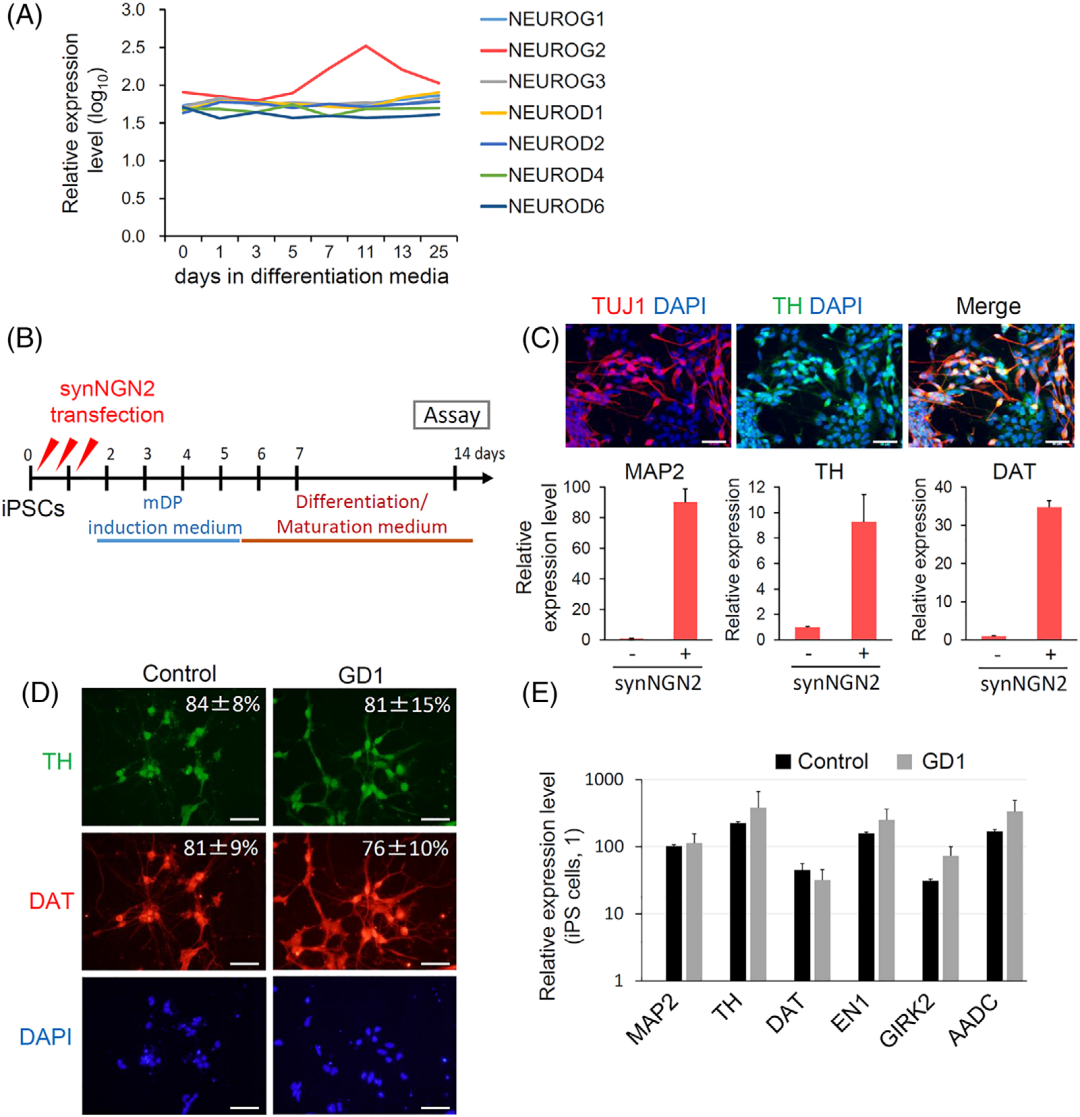
Direct neuronal differentiation of patient induced pluripotent stem cells (iPSCs) by the RNA‐based method. A, Expression changes in transcription factors (TFs) related to neuronal induction during the differentiation of iPSCs into dopaminergic (DA) neurons induced by the previous method. Microarray data^30^ were used for this analysis and expression levels were calculated by ExAtlas.^33^ B, Experimental scheme of the RNA‐based method for iPSC differentiation into DA neurons. Synthetic RNA encoding NGN2 (synNGN2) was transfected three times during the first 2 days. The cells were cultured in mDP induction medium for 4 days and further cultured in differentiation/maturation medium for 9 days. Some of medium components are different from Figure 1A. The details are shown in Section 2. Immunostaining and RT‐PCR analyses were performed at day 14 of differentiation. C, Immunostaining analysis for TUJ1 and tyrosine hydroxylase (TH) in neurons induced from 201B7 iPSCs. DAPI, nuclei. Scale bar, 50 μm. Quantitative real‐time PCR analysis for neuronal markers in the induced neurons with synNGN2 or without synNGN2 transfection. MAP2 for matured neurons, TH and DAT for DA neurons. D, Immunostaining analysis for DA neuron markers, TH and DAT in the neurons induced from Gaucher disease type 1 (GD1) patient iPSCs and control iPSCs. Scale bar, 50 μm. Percentages of TH+ cells/DAPI+ cells and DAT+ cells/DAPI+ cells are shown. Mean ± SD (N = 3). E, Quantitative real‐time PCR analysis for neuronal markers in the neurons induced from GD1 patient and control iPSCs. MAP2 for matured neurons, TH, DAT, EN1, GIRK2, and AADC for DA neurons

Above experiments revealed that GlcCer is accumulated only in neurons at the late maturation stages. Therefore, we tested whether synRNA‐based TF introduction can accelerate the accumulation of GlcCer. Among TFs related to neuronal induction, we selected NEUROG2 (also known as NGN2) as a TF, because it is transiently expressed during iPSC differentiation using the conventional method (Figure 2A), and also it is known that NGN2 is required for the development of midbrain DA neurons.^34^ We used essentially the same culture conditions as reported in the conventional differentiation method^30^; however, to match the accelerated differentiation by synRNA, the overall culture period was shortened and the NPC expansion phase was omitted, as we have shown that a synRNA directly differentiates iPSCs into mature neurons^25^ (Figure 2B, see also details in Section 2). In addition, the differentiating cells were treated with aphidicolin, an inhibitor of DNA synthesis, to prevent the proliferation of undifferentiated cells or nonneuronal cells, resulting in the culture with mostly neurons. After NGN2 induction, morphological changes into neurons occurred in 5 days as shown previously.^25^ At 15 days of differentiation, neurons expressed dopaminergic markers, TH and DAT as well as general neuron makers, TUJ1 and MAP2 (Figure 2C,D). No differences were observed in the expression levels of these markers between GD1 patient and control cells (Figure 2D,E).

### 3.3 Accumulation of GlcCer in directly differentiated neurons from GD1 patient iPSCs

**FIGURE 3:**
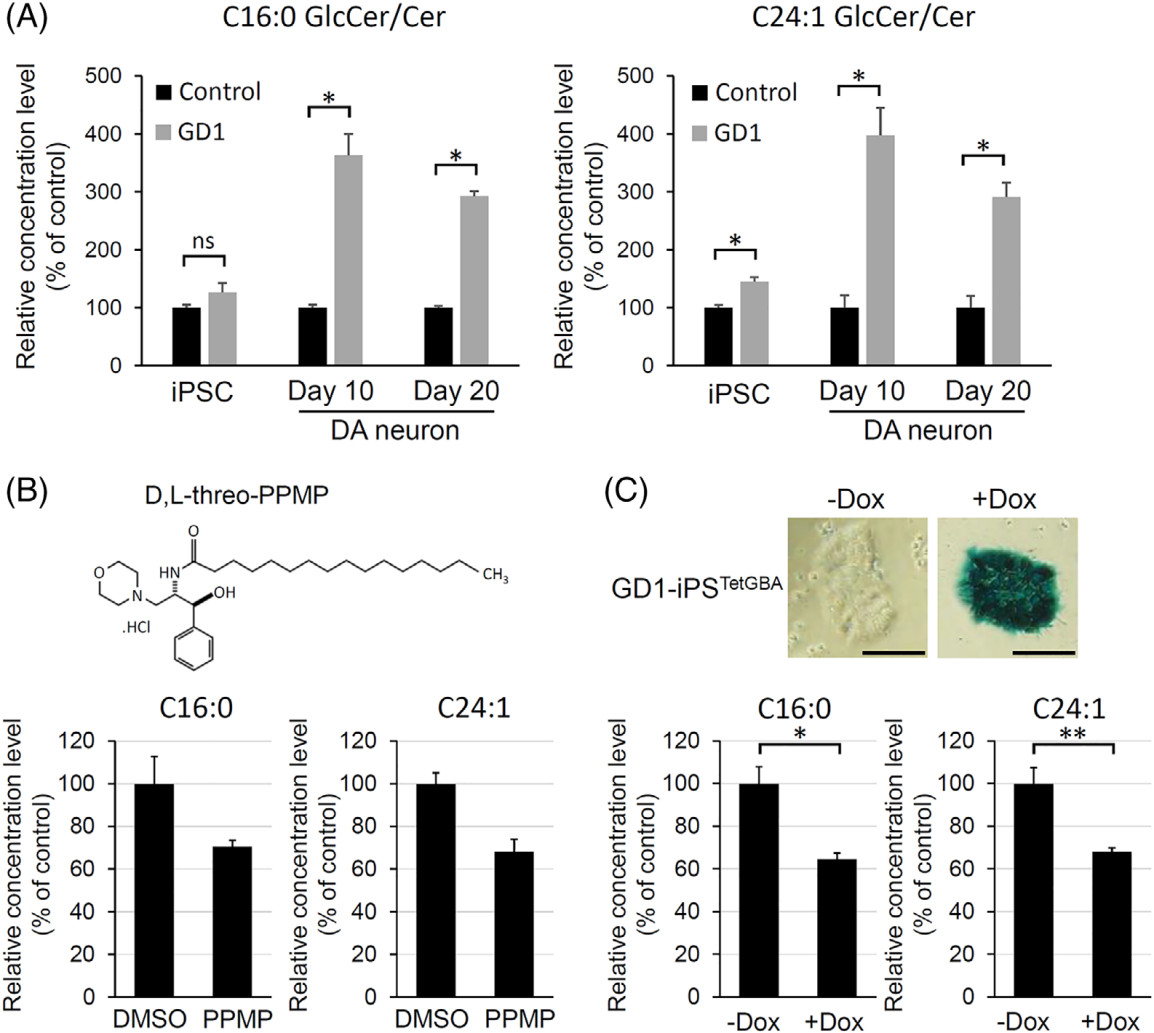
Accumulation of glucosylceramide (GlcCer) in directly differentiated neurons. A, liquid chromatography‐mass spectrometry (LC/MS) analysis for GlcCer concentration during DA neuronal differentiation by the RNA‐based method. Two GlcCer isoforms (C16:0 and C24:1) were analyzed. The ratios of GlcCer:Cer are shown. Induced pluripotent stem cells (iPSCs) (day 0) and DA neurons at day 10 and day 20 of differentiation were used for LC/MS. GD1, Gaucher disease type 1 patient cells; control, 201B7 cells. N = 3. *P < .01, *t* test. B, LC/MS analysis for GlcCer/Cer ratios in induced neurons treated with or without d,l‐PPMP. d,l‐PPMP (2 μg/mL) was treated during the differentiation of GD1 patient iPSCs. Cells were treated with dimethylsulfoxide (DMSO) as a control. N = 2. C, Tet‐on inducible iPSC line in which wild‐type GBA1 is expressed by doxycycline treatment (GD1‐iPS^tetGBA^) was generated as previously described.^29^ Doxycycline (Dox) was used to induce GBA1 expression during the differentiation. X‐gal staining was used to confirm GBA1 expression. Scale bar, 100 μm. LC/MS analysis showed that the GlcCer/Cer ratios were reduced by the wild‐type GBA. N = 3. *P < .01, **P < .05, *t* test

Using the synRNA‐based direct differentiation protocol, we examined GlcCer accumulation in DA neurons derived from GD1 iPSCs. LC/MS analysis was performed at day 0, day 10, and day 20 of differentiation. In addition, we measured the concentration of ceramide (Cer), because the ratio of GlcCer:Cer is correlated with disease severity.^35^ At the undifferentiated state (day 0), there was little difference in the GlcCer/Cer ratios (both C16:0 and C24:1) between GD1 and control cells (Figure 3A). However, at day 10, when most cells were differentiated into DA neurons, the GlcCer/Cer ratios (both C16:0 and C24:1) were significantly higher in GD1 cells compared with control cells (Figure 3A). Subsequently, the higher ratios of GlcCer/Cer (both C16:0 and C24:1) in GD1 cells compared with control cells were maintained until day 20. Furthermore, the accumulation of GlcCer (both C16:0 and C24:1) was inhibited by treatment with d,l‐threo‐PPMP, an inhibitor of GlcCer synthesis (Figure 3B), suggesting that the metabolism of GlcCer is dysregulated in GD1 patient‐derived neurons. To test whether GBA1 mutations is directly involved in the GlcCer accumulation, wild‐type GBA1 was exogenously expressed during the differentiation of GD1 patient iPSCs (Figure 3C). We found that the concentration of GlcCer was significantly reduced by the expression of a wild‐type GBA1 (Figure 3C), confirming that the neuronal accumulation of GlcCer in GD1 is caused by GBA1 mutations.

### 3.4 Phosphorylation of α‐synuclein in directly differentiated neurons from GD1 patient iPSCs

**FIGURE 4:**
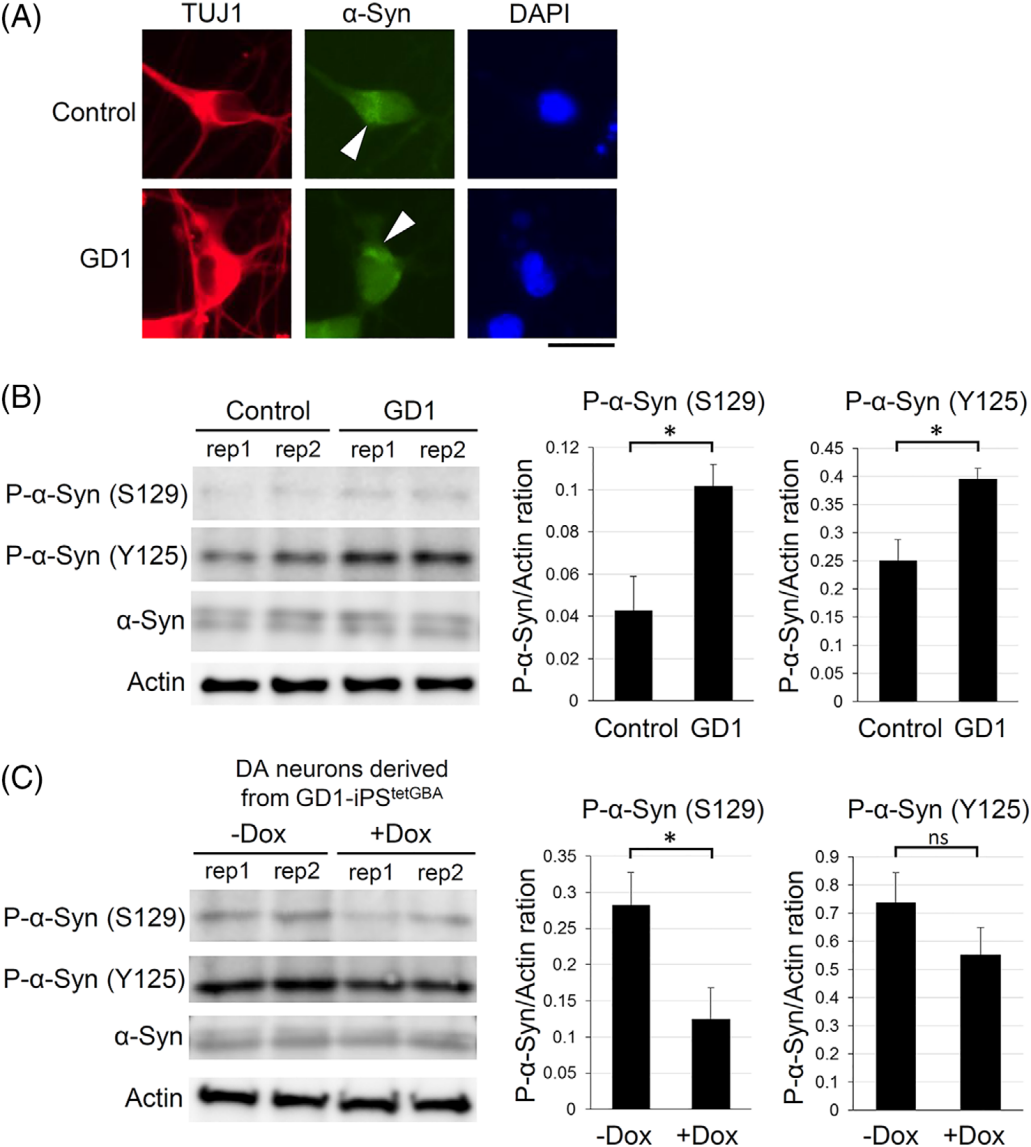
Phosphorylation states of α‐synuclein in directly differentiated neurons. A, Immunostaining analysis for α‐synuclein (α‐Syn) in the neurons from Gaucher disease type 1 (GD1) patient‐induced pluripotent stem cells (iPSCs) and control 201B7 iPSCs. TUJ1, neuronal marker. DAPI, nuclei. Arrowheads indicate intense α‐Syn staining. Scale bar, 20 μm. B, Immunoblotting analysis for phosphorylated α‐synuclein (P‐α‐Syn) in neurons from GD1 patient iPSCs and control iPSCs. Antibodies against phosphorylation at the S129 and Y125 were examined. Total α‐Syn and β‐actin were used as loading controls. rep1 and rep2 indicate biological replication. The right graphs show the quantification of phosphorylated α‐synuclein normalized to β‐actin. The average signals were obtained from three independent biological replicates. *P < .05, *t* test. C, Immunoblotting analysis for phosphorylated α‐synuclein in the neurons derived from GD1 patient iPSCs in which wild‐type GBA1 was expressed. GD1‐iPS^tetGBA^ were differentiated by RNA‐based method with or without Dox treatment. rep1 and rep2 indicate biological replication. Right graphs show the quantification of phosphorylated α‐synuclein normalized to β‐actin. The average signals were obtained from three independent biological replicates. *P < .05, *t* test

GlcCer accumulation is considered as a cause of α‐synuclein misfolding and aggregation into Lewy bodies.^36^ To investigate α‐synuclein aggregation in GD1 neurons, we immunostained for α‐synuclein. Differentiated neurons showed some clustered staining of α‐synuclein. However, we could not find any differences in staining patterns between GD1 patient and control cells (Figure 4A). We then tried to semi‐quantify phosphorylation of α‐synuclein by immunoblotting. Phosphorylation at the serine 129 has been shown to promote neurotoxic α‐synuclein oligomerization.^14^, ^37^ In contrast, phosphorylation at the tyrosine 125 is reported to protect from the neurotoxicity.^37^ Phosphorylation of both sites increases the binding affinity of metal ions that can modulate α‐synuclein aggregation.^38^ Importantly, the phosphorylation states of these sites have not been determined in GD1 patient neurons. We found that phosphorylation is enriched at both residues in neurons derived from GD1 patient iPSCs compared with control cells (Figure 4B). Moreover, the higher levels of phosphorylation of both sites was reduced by the expression of wild‐type GBA1 (Figure 4C). These results suggest that reduced GBA1 activity is involved in the hyperphosphorylation of α‐synuclein in GD1 patient neurons.

## 4 DISCUSSION

In the current study, we utilized an RNA‐based iPSC differentiation method to detect early a risk factor of Parkinson's disease, i.e. the accumulation of GlcCer, in mature GD1 patient‐derived DA neurons. In addition, using this rapid differentiation method, we showed the phosphorylation of α‐synuclein—a critical Parkinson's disease‐linked phenotype, in GD1 patient neurons. Finally, we demonstrated that the observed abnormalities were reversed by GlcCer synthesis inhibition or wild‐type GBA1 overexpression.

We examined the accumulation of GlcCer using the conventional differentiation method. We found that GD1‐specific accumulation was not detected until the maturation stage (~60 days of differentiation), as undifferentiated iPSCs and immature neurons did not show significant differences in GlcCer concentration between patient and control cells. These results suggest that GBA1 mutations do not affect GlcCer metabolism at early differentiation (or embryonic) stages and neuron‐intermediate stages. Consistently, we did not find significant defects in growth and differentiation of GD1 patient iPSCs. While the GBA1 mutation underlying GD1 causes reduced enzymatic activity, it may be enough to degrade GlcCer at the normal levels during early differentiation. Alternatively, GBA1 may not contribute to GlcCer degradation during the early stages of development. The cell‐type specific mechanisms may involve in the GlcCer metabolism. Stem cells have the distinct sphingolipid biosynthesis and metabolic pathways,^39^, ^40^ which might result in efficient GlcCer degradation without the need for high enzymatic activity. Furthermore, in neurogenesis, GlcCer synthesis is involved in axonal elongation, synaptic transmission, and neuron‐glia interaction,^41^, ^42^, ^43^ suggesting that GlcCer synthesis actively occurs at the later stages of neurogenesis. Excessive levels may be present in mature neurons due to defects in the balance of GlcCer synthesis and GBA1 mutation‐mediated degradation.

Abnormal GlcCer accumulation was detected earlier with synRNA‐NGN2 transfection (10‐15 days) than the conventional method. Induced expression of NGN2 has been reported to enhance neuronal induction of iPSCs and is, thereby, commonly used for generating specific neuron types, such as glutamatergic neurons and motor neurons.^25^, ^44^, ^45^ In this study, using the conventional differentiation method, we observed that endogenous NGN2 is transiently expressed during DA neuronal differentiation. It has been shown that Ngn2 is expressed in mouse ventral midbrain and required for the development of DA neurons.^34^ Although the functional role of NGN2 in the differentiation in vitro has not been clarified, recent studies reported that forced NGN2 expression enhances DA neuronal differentiation through synergistic activity with other neuronal induction TFs, ATOH1^46^ or Nurr1.^47^ This suggests that NGN2 expression is required for neuronal induction rather than the specification. The RNA‐based protocol followed previously published conventional differentiation conditions, as FGF8 and SHH were used as important factors for DA neuron specification.^19^, ^48^

Here, we showed that α‐synuclein is highly phosphorylated in GD1 patient‐derived neurons. Phosphorylation at S129 is linked with aberrant accumulation of α‐synuclein and neurotoxicity. It has been observed in the brain of patients with synucleinopathy, as well as animal models of Parkinson disease.^12^, ^13^, ^49^, ^50^ GBA1 deficiency is a possible cause of increased phosphorylation due to the reduced activity of protein phosphatase 2A (PP2A). PP2A is an enzyme that facilitates dephosphorylation of α‐synuclein.^51^ Loss of GBA1 function promotes α‐synuclein accumulation through autophagic defects accompanied by PP2A inactivation.^52^ Moreover, increases in GlcCer mediated by GBA1 deficiency lead to decreased ceramide production, which is known as an activator of PP2A.^53^ Thus, it is suggested that GBA1 mutants increase α‐synuclein phosphorylation at S129 via the inactivation of PPA2. In contrast to S129, phosphorylation at Y125 is regulated by other mechanisms and has opposing effects. In fact, in a Drosophila model, tyrosine phosphorylation was reported to suppress α‐synuclein oligomerization, thereby acting as a neuroprotectant.^37^ Our results showed that both residues are hyperphosphorylated in GD1 patient derived neurons. We speculate that phosphorylation at Y125 may antagonistically restrain the formation of the toxic α‐synuclein aggregation induced by phosphorylation at S129.

We previously showed that RNA‐based gene induction does not require the design of complex plasmid constructs, virus packaging, or the generation of stable cell lines.^23^, ^25^ The RNA‐based method enables the efficient and rapid translation of TFs.^25^ Therefore, synthetic mRNAs can be used for a simple and robust differentiation of iPSCs. To simplify the procedure, we omitted a replating step that is typically used for the purification of neurons, and instead added aphidicolin to suppress the proliferation of the residual nonneuronal cells. The advantages of rapid differentiation methods include a reduction in differentiation time as well as reductions in effort and cost, indicating their value for medical applications such as disease modeling and drug screening. In this study, we showed GlcCer accumulation caused by GBA mutations is observable within 15 days of differentiation. Furthermore, we showed that GlcCer accumulation can be suppressed by inhibiting GlcCer synthesis. Finally, our method demonstrated that the phosphorylated α‐synuclein and its dysregulation by GBA1 mutations can be assessed without the long‐term cell differentiation procedure.

In conclusion, we demonstrated that the RNA‐based method enabled us to rapidly perform biological and pathological investigations of neuronal diseases. This differentiation method could be used for a variety of applications, such as rapid evaluation of pathological differences between patients and high‐throughput screening of new therapeutic targets and drug discovery.

## CONFLICT OF INTEREST

O.S., Sh.S., M.S., H.N., and H.I. are employees and receive salary from Takeda Pharmaceutical Company, Limited. M.S.H.K. is a founder of Elixirgen Scientific, Inc., who receives a patent license from the Keio University and commercializes transcription factor‐based human pluripotent stem cell differentiation reagents. The other authors declared no potential conflicts of interest.

## AUTHOR CONTRIBUTIONS

T.A., H.I.: conceived and supervised the project, performed the experiments and analyzed the data, wrote the manuscript with input from all authors; Sa.S., O.S., Sh.S., M.S., H.N.: performed the experiments and analyzed the data; S.B.H.K.: contributed to the data acquisition, supervised the project; M.S.H.K.: supervised the project, wrote the manuscript with input from all authors.

## Data Availability

The data that support the findings of this study are available from the corresponding author upon reasonable request.
